# Current status of plant metabolite-based fabrication of copper/copper oxide nanoparticles and their applications: a review

**DOI:** 10.1186/s40824-020-00188-1

**Published:** 2020-06-03

**Authors:** Khwaja Salahuddin Siddiqi, Azamal Husen

**Affiliations:** 1 Department of Chemistry, Aligarh Muslim University, Aligarh, Uttar Pradesh 202002 India; 2 Wolaita Sodo University, P.O. Box: 138, Wolaita, Ethiopia

**Keywords:** Cu/CuO NPs, Biogenic synthesis, Biomedical, Crop growth, Applications

## Abstract

Since green mode of nanoparticles (NPs) synthesis is simple, advantageous and environment friendly relative to chemical and physical procedures, various plant species have been used to fabricate copper and copper oxide nanoparticles (Cu/CuO-NPs) owing to the presence of phytochemicals which often act as capping as well as stabilizing agent. These Cu/CuO-NPs are highly stable and used in the degradation of organic dyes like methylene blue and reduction of organic compounds such as phenols. They are also used as antibacterial, antioxidant and antifungal agent due to their cytotoxicity. They are also examined for agricultural crops growth and productivity. Cu-NPs increased the root and shoot growth of mung bean. In wheat plants, these particles reduced shoot growth; and enhanced the grain yield and stress tolerance through starch degradation. Similarly, CuO-NPs treated seedlings have shown reduced chlorophyll, carotenoid and sugar content, whereas proline and anthocyanins were increased in *Brassica rapa* seedlings. Overall, this review presents the recent understanding of plant-mediated Cu and CuO-NPs fabrication and their application in biomedicine, environmental remediation and agricultural practices. A comparison of the traditional/conventional method of fabrication of NPs with those of green protocols has also been made. Some misconception of copper chemistry has also been critically discussed in terms of oxidation and reduction reactions.

## Introduction

**Fig. 1:**
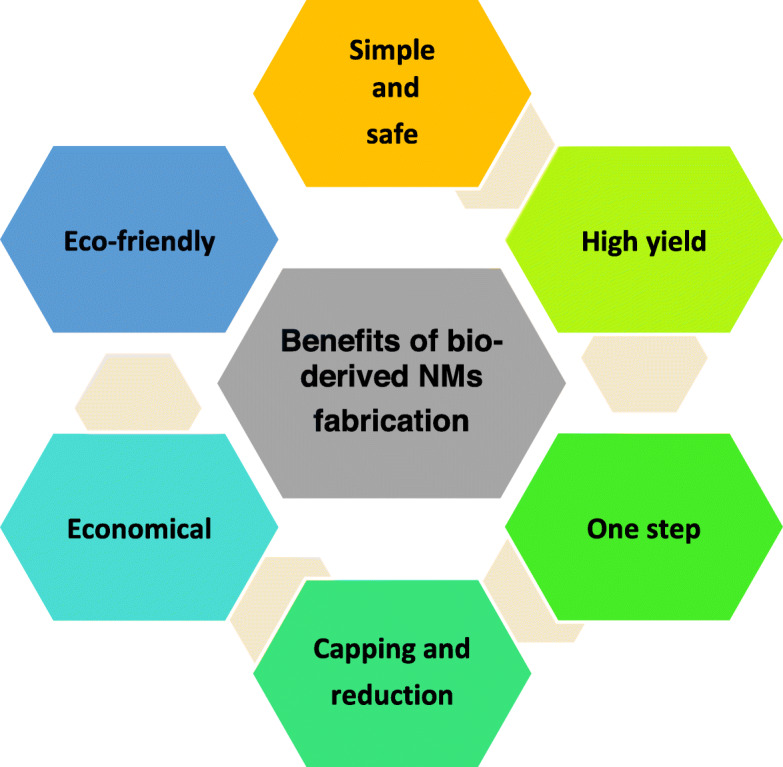
Benefits of bio-derived fabrication/green synthesis of nanoparticles over chemical and physical procedures

**Fig. 2:**
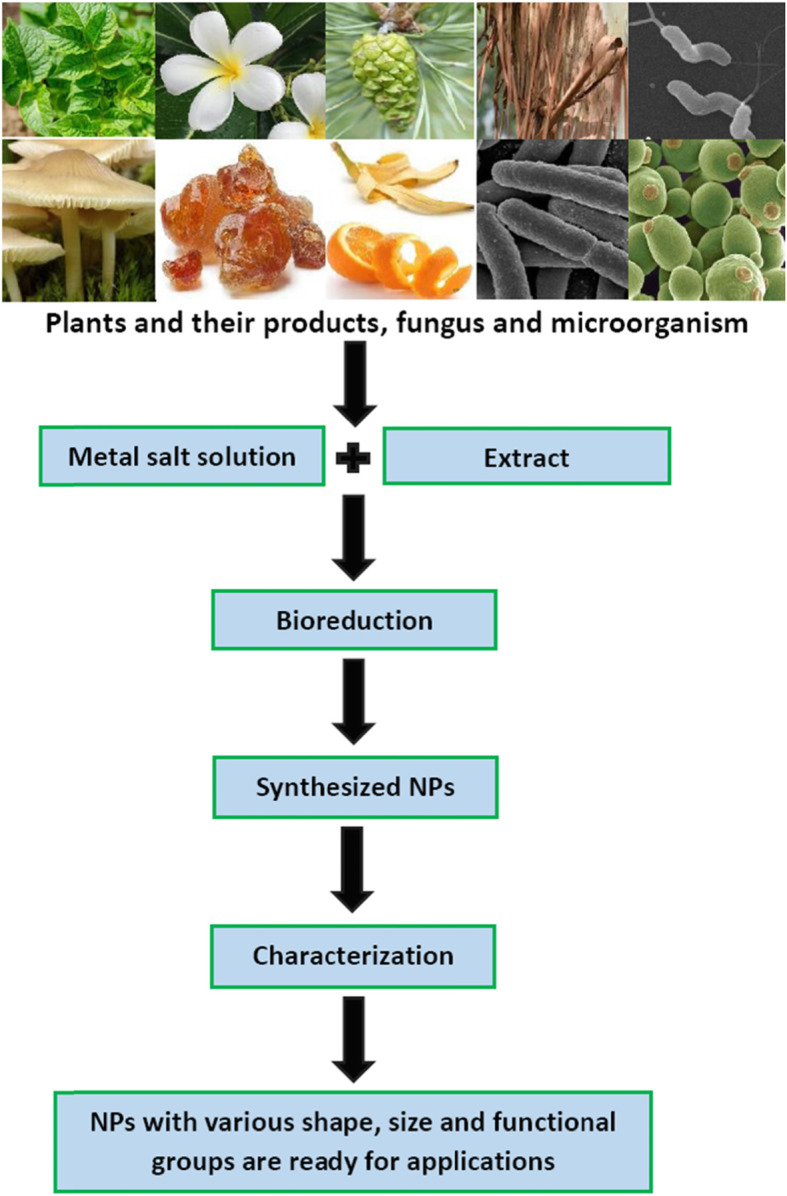
Detailed scheme of bio-derived fabrication/green synthesis of nanoparticlesusing lower/higher plant materials and their products; fungus and microorganisms

Recent advancement in nanotechnology has accelerated our interest in designing nano sized particles of desired shape and size. Since their property changes with morphology they have been used in various areas of medicine, agriculture and environmental remediation [1–4]. The ‘green synthesis’ of nanoparticles (NPs) using plant is advantageous over chemical, physical and or microbial synthesis as it removes the complicated protocol; and can also meet the large-scale production requirement. Further, the deliberate synthesis of NPs by chemical/physical methods require fairly large amount of toxic chemicals which also leave undesirable materials that pollute the environment. Disposal of such by products is also hazardous to human beings. Besides the use of expensive chemicals, the NPs thus fabricated are not capped/coated, and therefore they are not protected and are relatively less stable than those produced by plant-based materials. In order to protect these nanomaterials (NMs) they are coated with polyethylene glycol like polymers. Methods using lower and higher plant materials and their products; fungi and sometimes microorganisms for NPs fabrication are eco-friendly (Figs. 1 and 2) [2–5]. Since these materials are easily available and do not require organic solvent as reaction medium, they are easy to handle and economical. In major cases the NMs thus synthesized are capped by biomolecules like phenols, tannin, flavonoids and ascorbate present in the plant materials. They enhance stability of NPs and also prevent their interaction with atmospheric oxygen. These NMs are thus not oxidized and can be kept for long period of time without undergoing any change in their properties. Green synthetic methods make use of many waste materials like banana peels, lemon rind, dried leaves of medicinal plants and algae etc. The precursor even in crude form may react with these materials to produce NPs. The noble metal NPs for example silver, gold, platinum and their alloys have been frequently biosynthesized by various workers [6–12]. Among the coinage metal NPs, copper nanoparticles (Cu-NPs) are of great interest due to their low cost, easy availability and high electrical conductivity [13–15]. Borkow and Gabbay [16] and Zheng et al. [17] have reported that the copper ions are used as pesticides, fungicides and fertilizers. Copper oxide (CuO) is also a cheaper material in comparison to silver/gold and can easily be mixed with polymers due to their stability [18]. Recently, Din et al. [19] have discussed the synthesis and characterization of cupric-oxide nanoparticles (CuO-NPs) together with their application as antioxidant, antibacterial and antifungal agent.

In general, Cu-NPs are used as an antimicrobial, antioxidant, antidiabetic, anti-inflammatory and antifouling agent when integrated in coatings, plastics and textiles [20–23]. They also find application in heat transfer fluids, sensors, e-sensitized solar cells, lithium ion batteries, gas -sensing, heterogeneous catalysis and as anticancer agent [24–28].

Based on the current information, the present review unfolds the protocol of plant-mediated bio-fabrication of Cu-NPs/CuO-NPs followed by their characterization and application in different areas. Attempt has also been made to enlist the impact of NPs on crops as growth promoter and the effect of size of NPs on their efficiency as catalyst and antibacterial agent.

## Fabrication of cu-NPs/CuO-NPs

### Cu-NPs

Synthesis of spherical Cu-NPs of 23 ± 1.10 nm from aqueous flower extract of *Millettia pinnata* and their characterization by ultraviolet–visible spectroscopy (UV-Vis), scanning electron microscopy (SEM), transmision electron microscopy (TEM), x-ray diffraction (XRD), Fourier transform infrared spectroscopy (FTIR) and selected area electron diffraction (SAED) has been reported by Thiruvengadam et al. [21]. UV-Vis study has confirmed the reduction of copper acetate to Cu-NPs. The maximum absorption was recorded at 384 nm which confirmed the surface plasmon resonance (SPR) of the NPs. Their FTIR spectrum indicated the presence of proteins, acids, flavonoids, polyphenols, carboxylic acid and alkaloids which reduced the copper ions into Cu-NPs.

Cu-NPs of 50–250 nm diameters have been fabricated from *Magnolia kobus* leaf extract at room temperature [29]. Most of these NPs were spherical in shape and their formation was confirmed from UV-Vis spectrum exhibiting a peak at 560 nm. As the concentration of Cu-NPs increases the intensity of absorption peak measured at 560 nm also increases. It has also been observed that with increase in temperature yield of the Cu-NPs increases. However, complete conversion was achieved at about 90–95 °C. It was observed that when concentration of *Magnolia* leaf extract was increased up to 20%, smaller and spherical NPs were formed [30]. It is suggested that organic molecules/metabolites acting as capping agents also cause aggregation of NPs but it takes long time. Energy-dispersive x-ray (EDX) spectra showed signals for copper along with oxygen and carbon. Signals for other elements may not be due to biomolecules adhering to the surface of Cu-NPs but they may be due to impurity present in the colloidal mixture of NPs and leaf extract. It has been noted that chemically fabricated NPs were air oxidized while those synthesized by *Magnolia* leaf extract were stable for more than 30 days [30]. It is because the biomolecules acting as capping agent protect the Cu-NPs from oxidation by air.

Kulkarni et al. [31] have examined the fabrication of Cu-NPs from copper sulfate and *Eucalyptus* sp. leaf extract at room temperature. A slight change in pH of the copper sulfate solution from 2.16 to 2.83 was observed when leaf extract at pH 6.96 was added followed by a change in colour of the resulting mixture which showed absorption at 572 nm in its Uv-Vis spectrum. FTIR spectrum of the Cu-NPs showed the presence of phenol, amine, amino acid and flavonoids. It has been suggested that the biomolecules present in the eucalyptus leaf extract act both as reducing and capping agent. The NPs were highly crystalline with face centred cubic (FCC) structure. Their average size was found to be 38.62 nm. It is worth observing that the concentration and pH of the reducing agent is mainly responsible for the production of NPs.

Biosynthesis of Cu-NPs from leaf broth of *Azadirachta indica* and the influence of concentration of precursor salt, concentration of leaf broth, temperature and pH of the medium were reported on the conversion rate of Cu-NPs [32] which are similar to those found above [31].

Ultrasmall Cu-NPsof 2.90 ± 0.64 nm diameter have been synthesized from lemongrass tea by one pot method [33]. Although, colour of the mixture containing both the Cu-NPs and lemongrass was yellow it did not exhibit any absorption in the UV-Vis region (350-950 nm). It has been ascribed to extremely small size of Cu-NPs which are devoid of surface plasmon resonance [34, 35]. It is strange and unusual for NPs to exhibit colour but no absorption. Generally, a sharp peak is observed in 560 to 570 nm region for Cu-NPs if they are larger than 5 nm [34–36]. Their IR spectra showed the presence of polyphenols, proteins and carbohydrate [37, 38] as reducing agents. SEM and TEM images showed the presence of finely divided and dispersed Cu-NPs. However, they were not found to be oxidized either to cuprous oxide or cupric oxide which is quite obvious because in presence of reducing agents oxidation cannot occur. It has been observed that polyethyleneglycol or polyvinyl pyr olidone can also prevent the NPs from oxidation and aggregation [39–41].

Cu-NPs synthesized using aqueous leaf extract of henna (*Lawsonia inermis*) showed an absorption peak at 570 nm which is the signature peak of CuO-NPs [42]. Blue copper sulfate absorbs at 810 nm but Cu-NPs are reddish brown if all copper ions have been reduced to NPs. It has been reported that Cu-NPs are surrounded by a thin film of copper oxide (CuO and Cu_2_O) which is characterized by absorption at 800 nm [43]. It may be due to the presence of already oxidized copper sulfate present as impurity because in solution it cannot be oxidized unless it is heated in open at about 900 °C. Cu-NPs are efficiently formed at pH 11. Perhaps reduction of copper ions is facilitated in highly alkaline medium as the lawsones are stabilized at higher pH. Electrical conductivity of calcined Cu-NPs was enhanced because all particles come closer as a result of which the mobility of electrons increases [44]. Authors have found that the absorption of Cu-NPsat 570 nm was lost and a new peak appeared at 285 nm when Cu-NPs were calcined. It is highly improbable for a calcined material to absorb in UV region of spectrum. It may be quite likely that the spectrum was not carefully run otherwise any transition metal or its oxide will not show any absorption in UV range. Their interpretation of electronic transition from inner to the outermost shell of copper [45] is highly unlikely because such situation occurs only when copper ion forms a coordination compound with a very strong ligand and forces one of the 3d^9^ electrons to be transferred to the outer 4d orbital. Since there are no copper ions this peak at 284 nm is most likely to be due to some impurity. SEM and EDAX showed agglomerated spherical Cu-NPs. The suggestion that Cu-NPs are oxidized to CuO on exposure to air is author’s lack of knowledge of copper chemistry. If the Cu-NPs are exposed to air they will form CuSO_4_ which is evidenced by a distinct blue colour on its surface but it is not oxidized to copper oxides.

Biogenic synthesis and characterization of Cu-NPs from *Citrus medica* juice has been reported [46]. UV-Vis spectrum showed absorption peak at 631 nm which has been taken as an evidence for the formation of Cu-NPs. Since the reduction reaction was carried out in an aluminum vessel instead of a glass vessel some fraction of aluminum would have dissolved in the citron juice. Blue CuSO_4_ solution turned reddish brown due to formation of Cu_2_SO_4_ after the addition of citron juice, followed by deposition of shiny brown precipitate on the wall of aluminum vessel. It is suggested that a more reactive metal displaces a less reactive metal from its compound in aqueous medium [47] according to the following reaction.

1 $$ 2{\mathrm{Al}}_{\left(\mathrm{s}\right)}+3{\mathrm{Cu}}_2{\mathrm{SO}}_4\left(\mathrm{aq}\right)\to \kern1.75em 6\mathrm{Cu}\left(\mathrm{s}\right)+{\mathrm{Al}}_2{\left({\mathrm{SO}}_4\right)}_3\left(\mathrm{aq}\right) $$

Also, it has been stated that usually, copper cannot be obtained through the reduction of CuSO_4_ in water [48]. The reduction stops at Cu_2_O stage due to aggregation of water molecules around copper core [49]. However, reduction of CuSO_4_ to Cu_2_O is highly improbable because it is unstable and quickly oxidized to CuSO_4_ again. Addition of a surfactant at this stage yields Cu-NPs. Size of Cu-NPs ranged between 10 and 60 nm although average size was found to be 33 nm.

Fabrication of Cu-NPs from aqueous leaf extract of *Euphorbia esula* has been reported [50]. Catalytic activity of the NPs for the reduction of 4-nitrophenol and ligand free Ullmann-coupling reaction has also been investigated. Phenols and flavonoids in the leaf extract of *E. esula* act as reducing and capping agent for Cu-NPs. It has been shown from TEM images and size distribution studies that Cu-NPs are of 20–110 nm diameter. However, particles with 40 nm diameter are in abundance with FCC structure. Catalytic activity of Cu-NPs for the reduction of 4-nitrophenol to 4-aminophenol has been studied in presence NaBH_4_. The reduction of nitrophenol to aminophenol was ascertained from a change in colour from light yellow to deep yellow followed by shift of absorption from 317 to 403 nm due to the formation of nitrophenolate ions. When Cu-NPs were added, the solution became colourless after 580 s with the appearance of a new peak at 300 nm, as a result of the formation of 4-amino phenol.

Fabrication of Cu-NPs of 15–20 nm from aqueous peel extract of *Punica granatum* has been achieved which also acts as reducing as well as capping agent [51]. The UV-Vis spectrum of Cu-NPs practically shows a flat line without any absorption in the vicinity of 500–700 nm. Although there is a very faint elevation at 585 it cannot be considered as an absorption corresponding to Cu-NPs [52]. The other absorptions below 400 nm have been assigned to the presence of proteins, enzymes and flavonoids. Since the mixture containing extract and CuSO_4_ was heated at 80 °C, the enzymes would have certainly denatured and therefore the absorption in the above region may be only due to other stable phytochemicals present in the extract.

Activated carbon microfibers (ACF) coated with copper and stabilized with sodium dodecyl sulphate have been used as carrier for copper transport [53]. It was calcined at 350 °C to CuO-ACF and finally reduced in a current of hydrogen to generate Cu-ACF carbon nanofibers (CNF). They were then grown on Cu-ACF to produce Cu-CNF/ACF [53]. The *Cicer arietinum* plant treated with Cu-CNF grew well relative to the untreated plant. Shoots of the plant showed the presence of Cu-CNF. Presence of copper and carbon fibers was seen in the shoot of plant. It is supposed to be translocated via xylem of the plant. This material is a source of transport of copper as a micronutrient. Cu-NP released is thought to be converted to Cu^2+^ ions and Cu-CNF is considered as growth stimulant to plants. Further details of various plant species and their parts used in Cu-NPs synthesis and other associated activities are presented in Table 1.

**Table 1:** Recent studies (during 2018–19) on plant-mediated synthesis of Cu-NPs, their morphology, and various applications

Plant	Part of plant used	Active compounds	Shape and size	Various applications	Key references
*Azadirachta indica*	Leaf	Flavonoids, terpenoids and polyphenols	Crystalline, cubical; ~ 48 nm	–	[32]
*Cissus arnotiana*	Leaf	Biomolecules	Spherical; 60–90 nm	Antibacterial and antioxidant activities	[121]
Green and black tea	Leaf	Flavonoids and phenols	Spherical; 26–40 nm	Aflatoxins adsorbent; antibacterial and antifungal activities	[122]
*Eichhornia crassipes*	Flower	Lawsone and phenols	Spherical; 12–15 nm	Detection of hazardous hydrogen peroxide	[123]
*Falcaria vulgaris*	Leaf	–	Spherical; 20 nm	Cutaneous wound healing potentials without any cytotoxicity; antioxidant, antifungal and antibacterial activites	[124]
*Millettia pinnata*,	Flower	Proteins, acids, favonoids, polyphenols, carboxylic acid and alkaloids	Spherical; 23 ± 1.10 nm	Antioxidant, antibacterial, antidiabetic and anti-inflammatory agents	[21]
*Persea americana*	Seed	Carboxylic acid acid alkanes	Spherical; 42–90 nm	Antibacterial, antifungal and antioxidant activities	[125]
*Quisqualis indica*	Floral parts	Alcohols and phenols	Spherical; 39.3 ± 5.45 nm	Suppression of B16F10 melanoma cell proliferation and inhibition of tumor growth	[95]
*Ziziphus spina-christi*	Fruit	Alcohols and phenols	Cubic type; FESEM:8–15 nm, XRD:8–15 nm	Nanoadsorbent and antibacterial activity	[96]

#### CuO-NPs

Biosynthesis of CuO-NPs from gum karaya has been reported [54]. A mixture of CuCl_2_ and gum in aqueous solution was made alkaline and heated at 75 °C with continuous stirring. Blue copper chloride turned black after 1 h which was separated as black powder. CuO-NPs were deposited on the surface of gum karaya perhaps due to their affinity for gum. XRD analysis showed the presence of crystalline CuO-NPs scattered all over the gum matrix which is identical to those reported earlier [55]. FTIR spectrum showed peaks in the lower region of the spectrum corresponding to Cu-O stretching frequency. It is difficult to distinguish between Cu-O of cupric oxide and those of cuprous oxide as the NPs may be a mixture of both the CuO and Cu_2_O. Since the gum used in this work is a naturally occurring bio-polymer it contains sugars and amino acids which act as reducing as well as capping agent for CuO-NPs. Also they remain adhered to the surface of the gum matrix through electrostatic force. Abboud et al. [56] have reported the fabrication of CuO-NPs from *Bifurcaria bifurcate* (brown alga) purely in aqueous medium. Formation of NPs was ascertained from a change in colour from dark blue →colourless→deep red→black. It was a slow process but on heating at 100 °C the reduction of Cu^2+^ to CuO-NPs was facilitated [57]. UV-Vis spectrum of CuO-NPs exhibited peaks at 260 and 650 nm. The former was assigned to cuprous oxide NPs and the latter has been attributed to cupric oxide NPs [58, 59]. Terpenoids present in the algal extract are responsible for the reduction ofCu^2+^to CuO-NPs. However, the peak observed at 260 nm in the UV-region cannot be assigned to the presence of cuprous oxide NPs because metal oxides (owing to their colour) can absorb only in the visible region of the spectrum. The colour of the colloidal mixture is neither purely black nor reddish. It is reddish black owing to the presence of both the cuprous and cupric oxide NPs which is also supported by uneven distribution and two crystalline phases corresponding to mono-clinic cupric oxide (CuO) and cubic cuprous oxide Cu_2_O NPs [45, 59, 60]. There were two types of NPs identified from TEM images. Spherical ones were in abundance along with some elongated CuO-NPs ranging between 5 and 45 nm diameters. Diterpenoids are the major components in alga which work as reductant and stabilizer for CuO-NPs [61].

**Fig. 3:**
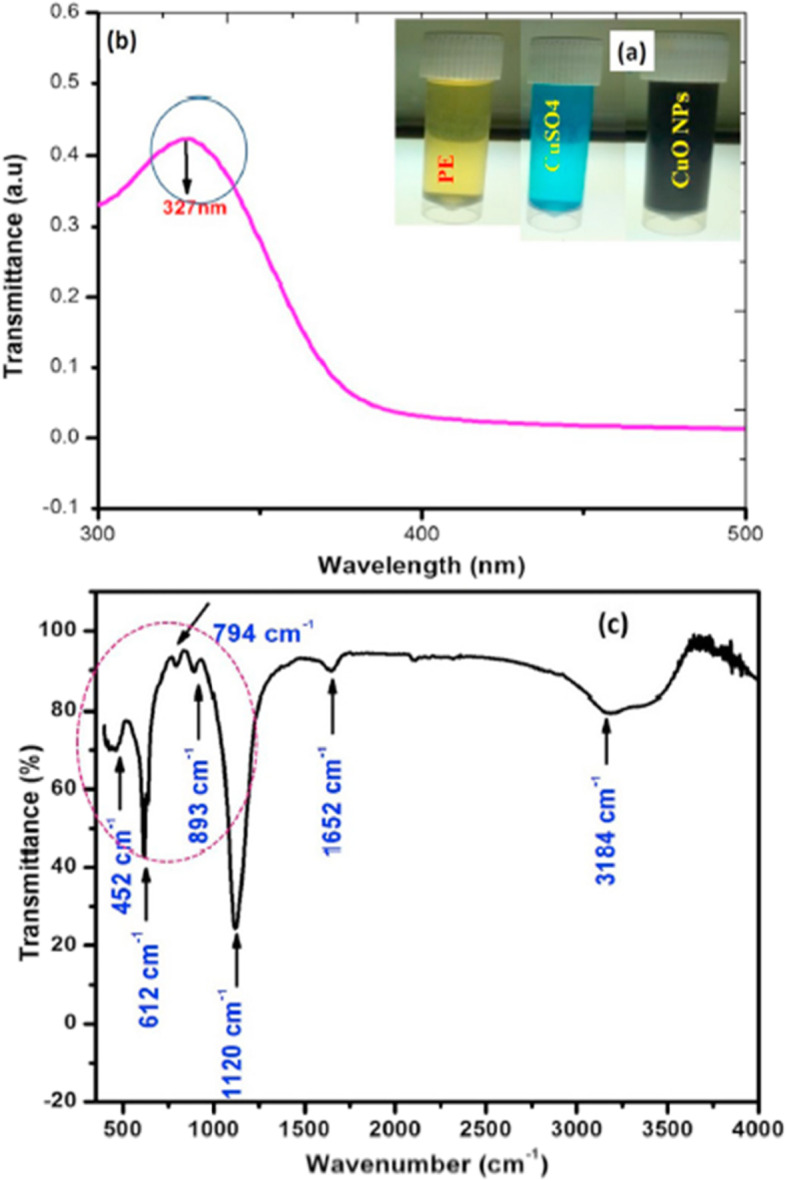
**a** Colouration of bio-fabricated CuO-NPs, **b** surface plasmon resonance UV-vis spectrum, **c** FTIR spectrum of CuO-NPs as fabricated from *Ruellia tuberosa *aqueous extract [64]

Fabrication of CuO-NPs from leaf extract (*Aloe vera*) by Vijay Kumar et al. [62] were shown to be monoclinic with an average particle size of 20 nm. Vishveshvar et al. [63] have shown CuO-NPs (obtained from *Ixoro coccinea* leaf extract) of an average size of 300 nm because of the formation of NP clusters. CuO-NPs obtained from *Ruellia tuberosa* aqueous extract were characterized by UV-Vis, FTIR, TEM, FE-SEM, EDAX and DLS [64]. In this experiment, the aqueous copper sulphate solution was added to the leaf extract showing a pale yellow to brownish black colour which had indicated the formation of CuO-NPs Fig. 3a). The absorption peak at 327 nm was confirmed by SPR (Fig. 3b) and also the formation of CuO nanorods of ~ 83.23 nm. Further, the FTIR studies have shown the characteristic peaks at 452 cm^− 1^, 612 cm^− 1^, 794 cm^− 1^, 893 cm^− 1^, 1120 cm^− 1^, 1652 cm^− 1^ and 3184 cm^− 1^ in 500–4000 cm^− 1^ range (Fig. 3c). Bands at 3184 cm^− 1^exhibited O − H stretch due to carboxylic acids, those at 1652 cm^− 1^established − C=C− bending due to alkenes. Strong bands at 1120 cm^− 1^exhibited the presence of C − O stretch due to alcohols and esters. The peak at 893.62 cm^− 1^ has been assigned to C − H bending due to aromatic groups. Strong peaks at 452 cm^− 1^, 612 cm^− 1^ and 794 cm^− 1^ revealed the presence of Cu − O vibrations in the fabricated CuO-NPs. Vasantharaj et al. [64] have suggested that the FTIR investigation had confirmed the availability of carboxylic acids, alkenes, esters, alcohols and aromatic compounds in the bio-fabricated CuO-NPs. Fabrication of CuO-NPs from leaf extract of *Psidium guajava* has been reported by Singh et al. [65]. In this investigation, CuO-NPs of different shapes and size ranging from nanorods to nano spheres were found.

CuO-NPs from *Eichhornia crassipes* weed has been successfully synthesized in aqueous medium under laboratory conditions [66]. The colloidal solution containing CuO-NPs and weed extract showed absorption at 310 nm in UV-Vis spectrum. SEM and TEM images showed spherical shaped CuO-NPs of approximately 28 nm. It is interesting to note that CuO-NPs were free from impurity which is confirmed from EDX pattern which showed peaks only for copper and oxygen.

Ghidan et al. [67] have also investigated the biosynthesis of CuO-NPs from aqueous peel extract of *Punica granatum* at room temperature. It has been observed that peel extract contains amines amino acids and phenols which reduce copper ions to CuO-NPs. Crystalline and spherical NPs of nearly 40 nm size were found to be agglomerated. SEM images showed particles of 10–100 nm. Toxicity of CuO-NPs was investigated against the mortality of green peach Aphids at different concentrations. Maximum mortality was observed at 8000 μg/ml which is quite significant.

Sivaraj et al. [68] have synthesized CuO-NPs from *Tabernaemontana divaricate* leaf extract in water. They were obtained in good yield as brownish black powder and identified from a peak between 220 and 225 nm in UV-Vis spectrum. Presence of phenol, amine and proteins has been suggested on the basis of IR spectral peaks. Spherical CuO-NPs of 48 ± 4 nm appear to be in pure state as the EDX showed sharp signals only for copper and oxygen [69]. These NPs inhibit the growth of *E. coli* at a very low dose of 25 μgml^− 1^. It is more than twice as effective as tetracycline.

CuO-NPs from dried aqueous fruit extract (*Tribulus terrestris*) has been shown to contain carbonyl and hydroxyl groups in the fruit extract [70]. Although many other compounds are suspected to be present in the extract the IR spectrum exhibited the presence of a single component. CuO-NPs of 5–22 nm size were dispersed nicely and biological molecules in the extract appear to be deposited on them.

Aqueous leaf extract of *Pterospermum acerifolium* has been used to synthesize CuO-NPs from hydrated copper nitrate [71]. NPs were oval shaped and pure as evidenced from EDX. They were more stable than the engineered NPs. However, hydrodynamic diameter of plant mediated CuO-NPs increased from 212 ± 47 to 634 ± 40 nm, whereas the particle size of engineered NPs increased up to 1037 ± 171 nm after 72 h. This property of increasing the hydrodynamic diameter may be used to control the size of CuO-NPs in aqueous medium. Perhaps the green synthesized NPs are capped by the phytochemicals present in the leaf extract and are therefore less aggregated. It has been noted that the rate of release of copper ions from engineered CuO-NP is much faster than plant synthesized NPs for identical concentration. It has been ascribed to stability and capping of plant synthesized CuO-NPs. It can be more effective and lasting if used in crop protection against pest and microbes as it is antibacterial in nature. Copper ion release is dependent on time, concentration and temperature to some extent [72, 73].

CuO-NPs and dissolved copper generally accumulate over the organisms exposed to them. Effect of NPs and free copper ions on *Elodea nuttallii* has been studied by Regier et al. [74]. CuO-NPs with large hydrodynamic size (1059 ± 88 nm) very quickly agglomerate and did not change even when irradiated with UV light. Uptake of CuO-NPs in suspension by *E. nuttallii* is less than the dissolved copper [75]. However, it is contradictory because large quantity of accumulated copper has been found in *Landoltia punctata* when exposed to CuO-NPs than when they were exposed to similar concentration of dissolved copper [76]. It has been argued that copper is solubilized from CuO-NPs due to acid exuded from growing shoots. However, copper ions would be available only if reduction of CuO-NPs occurs followed by its oxidation.

2 $$ \mathrm{CuO}+{\mathrm{H}}_2\mathrm{Redn}\kern0.75em \to \kern0.5em \mathrm{Cu}+{\mathrm{H}}_2\mathrm{O} $$

3 $$ \mathrm{Cu}\ \mathrm{Oxdn}\to {\mathrm{Cu}}^{2+}+2\mathrm{e} $$

It is obvious that more copper ions will be available from copper salts because it is highly ionized in water and can be transported to different parts of plants through osmosis. CuO-NPs get accumulated due to large size.

4 $$ {\mathrm{Cu}\mathrm{SO}}_4\leftrightharpoons {\mathrm{Cu}}^{2+}+2{\mathrm{e}}^{-} $$

CuO-NPs from *Gloriosa superb* leaf extract in water have been synthesized [77]. Spherical CuO-NPs of 8–17 nm were obtained in pure state as the XRD pattern shows single phase monoclinic structure although SEM images show particles of smaller size (5–10 nm).

Formation of CuO-NPs from *Carica papaya* leaf extract has been reported [78]. A sharp peak in the FTIR spectrum of colloidal cupric oxide NPs at 473 cm^− 1^ has been taken as an evidence for its formation. Presence of cuprous oxide NPs has been eliminated due to absence of any characteristic peak around 605–660 cm^− 1^. Rod shaped CuO-NPs of 140 nm are crystalline and have FCC structure [79]. Larger particles (614 nm) are not monodispersed perhaps due to capping by the phytochemicals in the papaya leaf extract.

Leaf extract of *Calotropis gigantea* has also been used for the fabrication of CuO-NPs. Steriods and polyphenols in the extract reduce Cu^2+^ ions to CuO-NPs and also act as stabilizer for them. It has been stated [26] that oxygen of ester and phenols form metal chelate with copper ion. On heating the chelate complex, CuO-NPs are obtained. It is surprising that the authors have said that CuO-NPs are formed by the phytochemicals and pure NPs are obtained after heating at 400 °C. A metal complex can be formed by a metal ion only. Free metal in atomic state cannot form a complex. A chelate with phenol cannot be formed as it is a monodentate ligand and cannot form a ring. By heating copper nitrate at 400 °C, CuO-NPs cannot be obtained because oxidation of copper to CuO can occur only at 900 °C. CuO-NPs were already formed by the leaf extract, and there were no complex whatsoever. Such hypothesis is baseless. Heating at 400 °C will cause burning of any organic matter left in excess and shall leave soot or carbon as impurity. Spherical CuO-NPs of 20–30 nm were formed as the EDX showed peaks only for copper and oxygen separately. The NPs were not well scattered.

Natural phytochemicals are frequently used for the fabrication of metal and or metal oxide NPs. A facile synthesis of CuO-NPs from *Gundelia tournefortii* aqueous extract of has been reported [80]. It has been shown that Cu^2+^ to CuO-NPs conversion occurs after heating the mixture of plant extract and CuCl_2_ at 60 °C for 2 h. IR spectra of extract and NPs exhibited the presence of phenolic compounds absorbed on the surface of CuO-NPs [81, 82]. NPs are highly crystalline [80] and spherical. Authors have concluded from EDX pattern that the presence of oxygen refers to the oxidation of Cu-NPs when exposed to air. It is not true because the Cu and oxygen peaks are due to CuO-NPs.

CuO-NPs fabricated from the aqueous flower extract of *Anthemis nobilis* had FCC, crystalline structure [83]. These NPs were found to be useful for the synthesis of propargylamines in moderate yield. Vegetable peels have also been used for the synthesis of CuO-NPs [84]. Cauliflower (*Brassica oleracea*), potato (*Solanum tuberosum*) and pea (*Pisum sativum*) peel extracts yield CuO-NPs from CuCl_2._ 2H_2_O.This reaction was very slow as it took 24 h at 60 °C to yield NPs. All NPs obtained from the above sources were monoclinic ranging from 22.2 to 31.60 nm. Since IR spectra did not show any peak at 610 cm^− 1^ the presence of Cu_2_O was ruled out [85]. As the Cu_2_O is deep red it could have been easily noticed. Distinction between Cu_2_O and CuO can hardly be made on the basis of Cu-O stretching frequencies as they are very closely spaced. Morphology of CuO-NPs prepared from different vegetable wastes vary in size. Their catalytic activity is also different from each other due to their shape, size and concentration.

Mixed metal nanomaterials have also gained popularity due to their multidisciplinary application. Biofabrication of Pd/CuO-NPs from *Theobroma cacao* seed extract and their application as catalyst has been reported [86]. The Pd/CuO-NPs were highly stable because no change was observed even when it was left for 30 days. Antioxidants like epicatechin, catechin and their derivatives in cocoa seed extract act as strong reducing as well as capping agent. Pd has face centered cubic structure mixed with CuO-NPs of about 40 nm. Agglomeration is prevented due to capping of Pd/CuO-NPs. Presence of oxygen in EDS spectrum has been ascribed to oxidation of Cu NPs to CuO-NPs. When Pd/CuO does not contain free copper ions what is the need of oxygen for oxidation of CuO-NPs. However, oxidation of metallic copper in presence of water can take several hours nevertheless it would give CuSO_4_ with a characteristic blue colour. However, authors assumption needs verification. The mechanism proposed for the formation of metal NPs and their nucleation is not convincing. All the phenolic –OH groups cannot be oxidized even in multiple steps as the drainage of electron from it will make the ring unstable. If such nucleation occurs a metal cluster would be formed.

Seeds of many leguminous plants also contain fairly reasonable amount of proteins, phenols, flavonoids, alkaloids and amino acids [87–89] and hence they are used as reducing agent to synthesize metal NPs. Crystalline monoclinic CuO-NPs of 26.6 nm have been synthesized from black been extract and their anti-cancer activity against HeLa cells has been reported [90]. They were effective in a very low concentration range (0.5–1 mg ml^− 1^) and short duration of time (12–48 h). It has been ascribed to intracellular ROS generation in a dose dependent manner.

The mechanism suggested for the formation of CuO-NPs is beyond imagination and conceptually non convincing. Authors have argued that the precursor CuSO_4_ reacts with hydroxyl anion OH-, generated by the ionization of water molecules and eventually reduced by phytochemicals present in the seed extract. It must be clarified at this stage that:

(a) It is universally known that water is not ionized until acidified water is electrolysed and (b) Cu (OH)_2_ can be formed only if NaOH or requisite amount of NH_4_OH is added to a copper salt as shown below.

5 $$ {\mathrm{CuSO}}_4+2\mathrm{NaOH}\to \mathrm{Cu}{\left(\mathrm{OH}\right)}_2+{\mathrm{Na}}_2{\mathrm{SO}}_4 $$

6 $$ {\mathrm{CuSO}}_4+2\mathrm{NH}4\mathrm{OH}\to \mathrm{Cu}{\left(\mathrm{OH}\right)}_2+{\left({\mathrm{NH}}_4\right)}_2{\mathrm{SO}}_4 $$

However, CuSO_4_ remains ionized in aqueous medium or it may form hexaaquo copper complex, [Cu(H_2_O)_6_].

7 $$ {\mathrm{Cu}\mathrm{SO}}_4\leftrightharpoons {\mathrm{Cu}}^{2+}+{{\mathrm{SO}}_4}^{2-} $$

Free Cu^2+^ ion is then reduced by protein or polyphenol available in the black bean extract. Phenol is oxidized to phenolate ion which provides electron to Cu^2+^ ions to from CuO-NPs.

Bawadi [91] and Bobe et al. [92] have reported that black bean extract inhibits the proliferation of breast, colon liver and prostate cancer cell. Also, it does not interfere with the functioning of healthy cells [93, 94]. It is still not understood as to why some peel/plant extract yield Cu-NPs and some of them produce CuO-NPs from the same precursor. As such CuSO4 will produce Cu^2^+ ions in aqueous medium and reduced to Cu-NP by phytochemicals. For CuO-NPs, Cu^2^+ is to be reduced to Cu-NPs and subsequently oxidized to CuO-NPs. However, its whole chemistry is yet to be investigated. Further details of various plant species and their parts used in Cu-NPs synthesis and other associated activities are presented in Table 2.

**Table 2:** Recent studies (during 2018–19) on plant-mediated synthesis of CuO-NPs, their morphology and various applications

Plant	Part of plant used	Active compounds	Shape and size	Various applications	Key references
*Aloe vera*	Leaf	–	Octahedral; 24-61 nm	Photocatalytic activity	[126]
*Ferulago angulate*	Aerial part	–	Shell like sheet structure; 44 nm	Photocatalytic activity	[127]
*Galeopsidis herba*	Plant extract	Flavonoids and phenolic acids	Spherical; 10 nm	Antioxidant and catalytic activity	[128]
*Ixoro coccinea*	Leaf	Carboxylic acids, alkenes, esters, alcohols and aromatic compounds	Spherical; SEM: 300 nm; TEM: 80–110 nm	–	[63]
*Malus domestica*	Leaf	Carbonyl, methyl, saturated aliphatic and alkane/alkyl groups	Spherical and crystalline; 18–20 nm	DNA cleavage, antibacterial and antioxidant activities	[129]
*Psidium guajava*	Leaf	–	Spherical; 2–6 nm	Photocatalytic activity	[65]
*Stachys lavandulifolia*	Leaf	–	Less than 80 nm	Antifungal activity	[130]
*Terminalia belerica*	Fruits	–	Spherical; 9–14 nm	Antibacterial activity	[131]
*Ruellia tuberosa*	Leaf	Carboxylic acids, alkenes, esters, alcohols and aromatic compounds	Nanorods; 83.23 nm	Antibacterial and photocatalytic activities	[64]

## Application of Cu-NPs/CuO-NPs

**Fig. 4:**
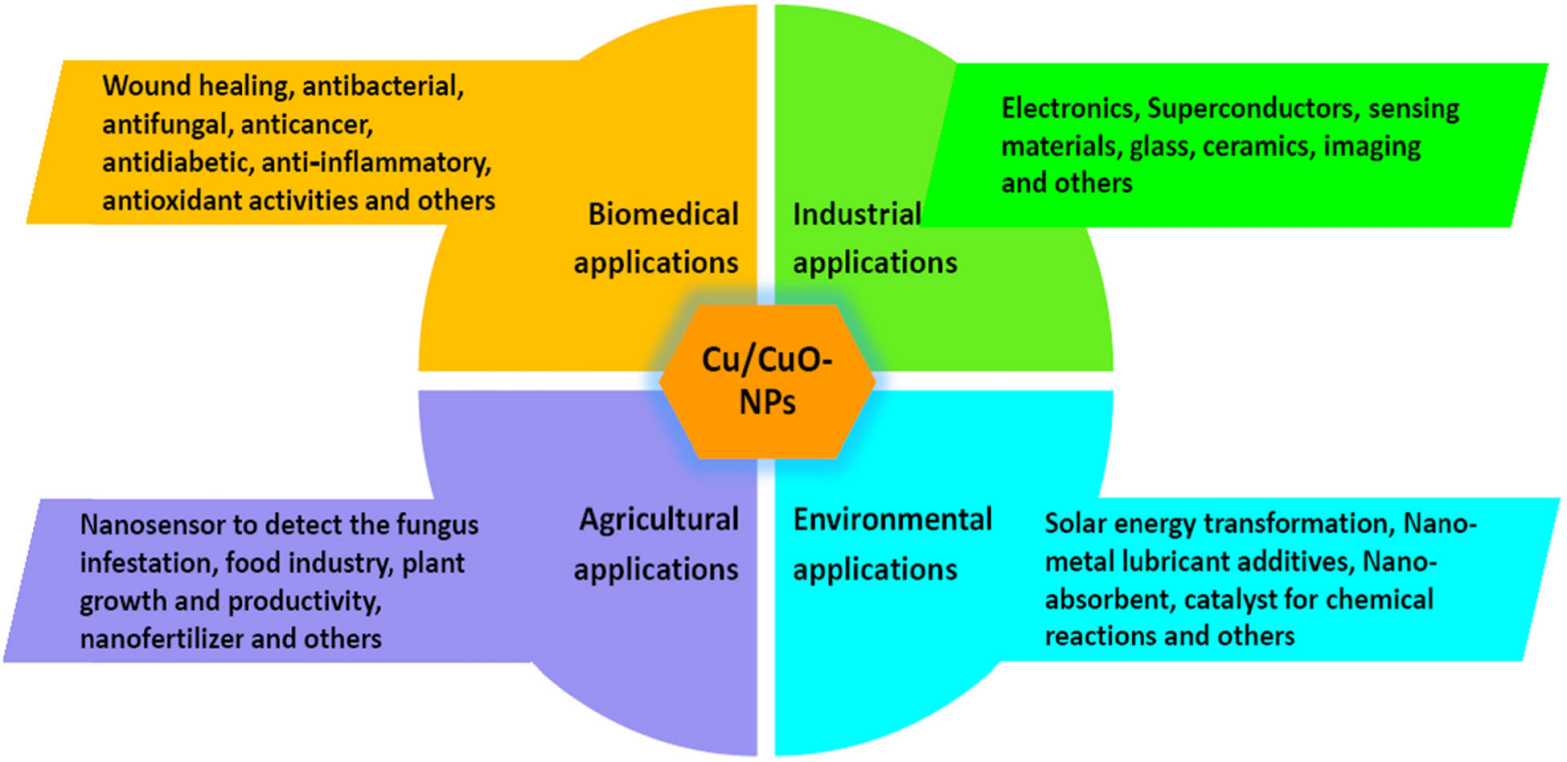
Application of Cu-NPs/CuO-NPs in biomedical, industrial, agricultural and environmental sectors

Application of Cu-NPs/CuO-NPs has been consolidated in Tables 1, 2 and Fig. 4.

### Antibacterial activity

Antibacterial activity of foam coated and uncoated Cu-NPswas screened. Biosynthesized Cu-NPs from *Magnolia kobus* aqueous leaf extract exhibited greater activity against *Escherichia coli* than those shown by chemically fabricated NPs [29]. Antibacterial activity of Cu-NPs synthesized from Citron juice (*Citrus medica*) was examined [49]. In vitro antimicrobial activity screened against *Escherichia coli*, *Klebsiella pneumoniae*, *Pseudomonas aeruginosa*, *Propionibacterium acnes* and *Salmonella typhi* showed that Cu-NPs were significantly effective against them. However, among the examined plant pathogenic fungi, *Fusarium culmorum* was observed to be most sensitive followed by *F. oxysporum* and *F. graminearum*. Cu NPs synthesized from *Punica granatum* peel extract have been found to be highly bactericidal against *Micrococcus luteus*, *Pseudomonas aeruginosa*, *Salmonella enterica* and *Enterobactor aerogenes* [51]. Their antibacterial efficacy was higher than that exhibited by standard antibiotic, streptomycin [97–99].. Biosynthesized Cu-NPs from *Millettiapinnata* flower extract of was examined against some pathogenic bacteria [21]*.* These particles were found to be more effective against *Staphylococcus aureus* and *Bacillus subtilis* (gram-positive) than *Pseudomonas aeruginosa* and *E. coli* (gram-negative).

CuO-NPs obtained from gum karaya showed very high antibacterial activity against *E. coli* and *S. aureus* [96]. Efficacy was found to be dependent on the size of NPs [100], the thickness of bacterial cell wall and incubation time. Smaller particles were more toxic than larger ones even if they do not penetrate the bacterial cell wall. Fabrication of CuO-NPs from *Bifurcaria bifurcate* have also been found to be significantly antibacterial against *Enterobacter aerogenes* and *Staphylococcus aureus* [56]. Bactericidal property has been attributed to the release of copper ions and their interaction with microbial cells. CuO-NPs obtained from an aquatic noxious weed, *Eichhorni acrassipes* have been found to act as growth inhibitor of *Fusarium culmorum* and *Aspergillus niger* [66].CuO-NPs obtained from the aqueous dried fruit extract of *Tribulus terrestris* were examined for their in vitro cytotoxicity and antibacterial activity [70].CuO-NPs also inhibit the growth of bacterial strains but they were found to be most effective against *Escherichia coli* [68]. SEM images of bacterial cells exposed to CuO-NP showed complete rupture of cell wall in 60 min which suggested that efficiency is time dependent. However, when bacteria were exposed to different concentrations of 2.5–100 μg/ml for about 1 h maximum decrease was found for lowest concentration. CuO-NPs obtained from *Gloriosa superba* leaf extract and examined for their antibacterial activity against *Klebsiella aerogenes*, *Pseudomonas desmolyticum*, *E. coli* and *Staphylococcus aureus* has been investigated [74]. CuO-NPs indicated significant activity against all bacterial strains but there is no conclusive study to propose a mechanism of action.

### Catalytic activity

Synthesis of Cu/RGO-Fe_3_O_4_ (Cu/Reduced graphene oxide-Fe_3_O_4_) nanocomposite from barberry fruit juice (*Berberis vulgaris*) and its application as a heterogeneous catalyst for the ortho arylation of phenols in presence of aryl halide has been reported [101]. It is interesting to note that nano composite was easily recovered and used several times without loss of catalytic activity. Since, barberry fruit juice contains mainly vitamin C and polyphenols, they act as reducing and capping agent for Cu-NPs. Although, CuO-NPs have been used in the hydration of phenylcyanamide to urea the yield was very small, it improved by increasing the temperature of reaction mixture. Reduction of 4-nitrophenol to 4-aminophenol by NaBH_4_ in presence of CuO-NPs has also been done. It takes only 70s for complete reduction but in absence of CuO-NPs the same reaction is not initiated even after 3 h. Fabrication and catalytic activity of Pd/CuO-NPs using *Theobroma cacao* was investigated through Heck coupling reaction and through reduction of nitrophenol to aminophenol [86]. The catalyst was reused up to 6 cycles. Mechanistic pathway is yet to be established. Cu-NPs from aqueous leaf extract of *Euphorbia esula* has also been used for catalytic reduction of 4-nitrophenol and ligand free Ullmann-coupling reaction [50].

The CuO-NPs fabricated from the leaf extract of *Psidium guajava* has exhibited a remarkable degradation efficiency against the industrial dyes. It degraded Nile blue by 93%, and reactive yellow 160 by 81% in just 120 min with apparent rate constants of 0.023 and 0.014 min^− 1^, respectively [65].CuO-NPs obtained from *Carica papaya* leaf extract has been exploited in the photocatalytic degradation of Coomassive brilliant blue dye in sunlight [78].

### Plant growth response

Plants are very susceptible to minor changes in the environment (abiotic stress) and supply of any trace element/NPs either as a nutrient or as a foreign matter [28, 102–108]. Cu-NPs enhanced the root and shoot growth of mung bean [109], but reduced shoot growth in wheat [110]. Yasmeen et al. [111] synthesized Cu and Fe-NPs and demonstrated their role on the growth as well as yield of wheat varieties. The metal NPs synthesized from onion extract of 15–30 nm diameters had irregular shape. Wheat varieties were treated with 20, 25, 30, 35 and 40 ppm Cu-NPs at different stages of growth until production of seeds. Spike length was invariably increased or remained unchanged when treated with 25 ppm Cu-NPs but significantly increased the number of grains. However, higher Cu concentration reduced the number of grains/spike and overall 1000 grain weight. Copper and iron NPs together at 25 ppm concentration have better effect on production and yield of wheat than copper or iron alone. Cu-NPs increased the grain yield and stress tolerance in wheat plant through starch degradation. Sugar content and SOD activity was enhanced in seeds treated with copper and iron NPs. Quantity of copper was increased while iron remained unchanged in seeds treated with Cu and Fe NPs [111].

ACF coated with copper and stabilized with sodium dodecyl sulphate have been used as carrier for copper transport [53]. It was calcined at 350 to CuO-ACF and finally reduced in a current of hydrogen to generate Cu-ACF carbon nanofibers. They were then grown on Cu-ACF to produce Cu-CNF/ACF [112]. The *Cicer arietinum* plant treated with Cu-CNF grew well in comparison to control plants.

Impact of engineered CuO-NPs on growth and development of *Arabidopsis thaliana* at molecular level has been explored [113]. Their seedlings were treated with different doses of CuO-NPs for 3 weeks. Plant biomass, total chlorophyll content and root elongation invariably decreased while an increase in anthocyanin, lipid peroxidation and proline were observed. Lignin was also found to be deposited in root. CuO-NPs treated seedlings also showed an increase in superoxide and hydrogen peroxide in leaves and roots which is directly proportional to its concentration. As a consequence of oxidative stress, the main root growth was inhibited but lateral root formation was initiated. This is mainly due to metabolic imbalance and as a response to resistance caused by CuO-NPs [114]. However, the plant system in response to such stress produces antioxidants to prevent the damage by ROS [104, 105, 115] which subsequently causes some changes in metabolic functions. Very recently, Chung et al. [116] have examined the effect of CuO-NPs on *Brassica rapa* seedlings. CuO-NPs treated seedlings have shown reduced root and shoot length, chlorophyll, carotenoid and sugar content, while proline and anthocyanins were increased. Additionally, production of malondialdehyde and hydrogen peroxide were increased in CuO-NPs treated seedlings which has been associated with DNA damage.

### Miscellaneous response

Recently, the biosynthesized Cu-NPs from flower extract of *Millettia pinnata* have also been shown to exhibit anti-diabetic and anti-inflammatory activities [21]. Human mesenchymal stem cells exposed to CuO-NPs at a concentration of 25 μg ml^− 1^ reduced their viability. CuO-NPs at a dose of or below10 μg/ml were non toxic to normal cells and therefore, a safe concentration may be used to prevent the interaction of CuO-NPs with normal mesenchymal cells. It has been suggested that CuO-NPs release Cu ions which are antibacterial and anticancerous [117, 118]. It also means that reduction of Cu-NPs to copper ions occur which are toxic to normal bacterial cells and cancer cells in mammals. Toxicity of CuO-NPs obtained from aqueous leaf extract of *Pterospermum acerifolium* was determined against *Daphnia magna* at several concentrations [71]. The engineered NPs were several times more toxic to *Daphnia* than green synthesized NPs [119, 120]. Release of copper ions from CuO-NPs is the main cause of toxicity to *Daphnia*. Dissolved copper ions from CuO-NPs get accumulated around *Daphnia* which cause toxicity.

## Conclusion

Plenty of nano particles are synthesized every year but only some of them are put to beneficial use. Green synthesized NPs of desired shape, size and stability can enhance their overall qualities for future applications. It appears that all plant species contain some type of phytochemicals which reduce the metal salts and metal oxides into their NPs. Cu and CuO-NPshave shown great promise as biocide and mild antibacterial agent. Even though, biofabrication of metal NPs from plants and microbes do not leave toxic residues in environment, their safe disposal is necessary. Further, it is also essential to utilize the fabricated Cu and CuO-NPs as biomedicine, environmental remediation and agricultural practices. Attention must be focused on their biocompatibility.

## Data Availability

Not applicable.

## Abbreviations

NPs: Nanoparticles
Cu-NPs: Copper nanoparticles
CuO-NPs: Cupric-oxide nanoparticles
UV-Vis: Ultraviolet–visible spectroscopy
SEM: Scanning electron microscopy
TEM: Transmission electron microscopy
XRD: X-ray diffraction
FTIR: Fourier transform infrared spectroscopy
SAED: Selected area (electron) diffraction
EDAX: Energy-dispersive x-ray spectroscopy
ACF: Activated carbon microfibers**.**
